# The Impact of Artificial Sweeteners on Human Health and Cancer Association: A Comprehensive Clinical Review

**DOI:** 10.7759/cureus.51299

**Published:** 2023-12-29

**Authors:** Wissam Ghusn, Roopa Naik, Marcel Yibirin

**Affiliations:** 1 Internal Medicine, Boston Medical Center, Boston, USA; 2 Medicine, Geisinger Commonwealth School of Medicine, Scranton, USA; 3 Internal Medicine/Hospital Medicine, Geisinger Health System, Wilkes Barre, USA; 4 Internal Medicine, Boston University School of Medicine, Boston, USA

**Keywords:** diabetes, side effects, health, cancer, artificial sweeteners

## Abstract

Artificial sweeteners are sugar substitutes that provide high sweetening power associated with low accompanied calories. In this study, we aim to review the data on the use, benefits, side effects, and cancer risks of artificial sweeteners. We reviewed data in the PubMed, MEDLINE, Google Scholar, Embase, and Scopus databases to search for studies about artificial sweeteners from the inception of the database to July 20, 2023, published in the English language. We discuss systematic reviews and meta-analyses, randomized clinical trials, and observational cohort studies that address the use of artificial sweeteners and their effect on health. In our review, we show that artificial sweeteners have been shown to impact various functions of the gastrointestinal system. Other studies have demonstrated an association with neurologic symptoms such as headache and taste alteration. Moreover, recent studies have established an association between artificial sweeteners and cardiovascular risk and diabetes. Importantly, the majority of research data show no link between the use of artificial sweeteners and cancer risk. Although most studies show that there is no established link between these products and cancer risk, artificial sweeteners are associated with multiple diseases. Hence, more studies are needed to better characterize the effect of artificial sweeteners on human health.

## Introduction and background

Artificial sweeteners (ASs), also known as high-intensity sweeteners, are sugar substitutes that provide high sweetening power associated with low accompanied calories [1,2]. Currently, there are six ASs approved by the Food and Drug Administration (FDA) [3]. These additives are known for their intense sweetness, often multiple times sweeter than sugar, allowing for smaller amounts to be used to achieve the desired level of saccharinity [1]. Their use has been beneficial in multiple fields, including weight and diabetes management [4], food and beverage sweetening [5], and oral health products and medicine [6].

The use of ASs has been constantly increasing in recent years. In the United States, it has been reported that almost 25% of children and more than 41% of adults have used ASs between 2009 and 2012 [7]. In addition, the cost of ASs reached approximately $2.2 billion in 2020 and is continuously expected to increase worldwide [8].

However, multiple studies have shown various side effects associated with the use of these sweeteners. These side effects include gastrointestinal symptoms [9], neurologic [10] and taste perception changes [11], allergic reactions [12], insulin and metabolic effects [13], and cardiovascular effects [14]. In addition, ASs have been shown to affect the gut microbiota that may mediate certain side effects [15]. Most importantly, many researchers have assessed the potential effect of ASs on the cancer risk of people who consume these products [16,17]. In this study, we aim to review the data on the use, benefits, side effects, and cancer risk of ASs. We used the following keywords in our search: “artificial sweeteners,” “sweeteners,” and “AS.” We included all articles that studied ASs without exclusions.

## Review

Methodology

In this clinical review, we reviewed data in the PubMed, MEDLINE, Google Scholar, Embase, and Scopus databases to search for studies about ASs from the inception of the database to July 20, 2023, published in the English language. We discuss systematic reviews and meta-analyses, randomized clinical trials (RCTs), and observational cohort studies that address the use of ASs and its effect on health.

Results

History, Benefits, and Uses

In 1879, Constantin Fahlberg, a chemist at the laboratory of Ira Remsen at Johns Hopkins University, discovered saccharin, which became the first commercially available AS [18]. It was accidentally synthesized while Fahlberg was working on coal tar derivatives. Later, in the early 20th century, more sweeteners were introduced to the market, including cyclamate and aspartame. However, serious carcinogenicity concerns were raised regarding cyclamate, resulting in an FDA ban during the 1970s [19]. Eventually, more sweeteners were consumed worldwide. As of 1974, six ASs were approved by the FDA as food additives, i.e., aspartame (1974), saccharin (1977), acesulfame potassium (1988), sucralose (1998), neotame (2002), and advantage (2014) (Table 1) [3].

**Table 1:** Food and Drug Administration (FDA)-approved artificial sweeteners, their sweetness compared to table sugar, common side effects, and associated cancer risk. *: Few studies suggest an association with cancer risk in rodents, but not humans. **: Sweetness compared to table sugar.

Artificial sweetener	FDA approval	Sweetness**	Cancer risk
Aspartame	1974	200	None*
Saccharin	1977	300	None
Acesulfame potassium	1988	200	None
Sucralose	1998	600	None
Neotame	2002	7,000–13,000	None
Advantame	2014	20,000	None

ASs have been used in various food industries for multiple uses and benefits. First, these sweeteners have been used in numerous areas in the food and beverage industry, including soft drinks, desserts, dairy products, coffee, and processed foods. These sweeteners aim to provide a sweet taste with minimal calories associated with sugar [5]. Second, AS provides medical benefits for weight management and in patients with diabetes mellitus. The lack of high caloric content of sugar allows patients to avoid the weight gain associated with sugar calories. Hence, patients who are overweight and obese benefit from these products to hinder further weight gain. In addition to weight management interventions, including lifestyle interventions and diet [20], anti-obesity medication [21], and bariatric procedures [22], ASs have been utilized to aid in achieving weight loss in patients who are overweight or obese [4]. ASs have also been used in diabetes diets to alleviate the regular spike in blood glucose after meals [23]. However, multiple studies have shown contradictory evidence of the positive effect of ASs on metabolic diseases and obesity [24]. Third, ASs have also been used in oral health products, including liquid medicine, cough syrups, and toothpaste. This has significantly enhanced the use of these products worldwide [6,25].

Role in Weight Management

AS have been increasingly used as healthier alternatives to sugar-sweetened products to curb the obesity epidemic. However, the evidence supporting their use for weight reduction or weight maintenance has been inconclusive. In a meta-analysis of 56 studies, of which 17 were RCTs, there was no statistically significant body weight change between adults given ASs and those given various sugars or a placebo [26]. However, a subgroup analysis of this study showed that consumption of ASs was associated with greater weight loss than consumption of caloric sweeteners or placebo. In another study [27], artificially sweetened beverage consumption was linked to an elevated body mass index, as noted in over 5,000 adults, followed for eight years, as well as an increase in abdominal obesity (measured by waist circumference) during the nine-year follow-up.

The increasing use of artificially sweetened beverages to replace water has also been extensively studied. In an RCT comparing 300 people who were overweight or obese [27], consuming over 24 ounces of artificially sweetened beverages led to a greater degree of weight loss compared to the cohort drinking the same quantity of water. In another RCT [28], the replacement of water with an artificially sweetened beverage led to increased weight loss at 12 months.

Patients who are planning to undergo bariatric surgery are often recommended a low-calorie diet to promote preoperative weight reduction and reduce the risk of surgical complications. In such instances, ASs have been used as flavor enhancers for low-energy foods [29].

Side Effects

Gastrointestinal: ASs impact various functions of the gastrointestinal system, including the gut microbiome, gastrointestinal motility, intestinal absorption and permeability, and the anatomy of the gastrointestinal tract [9].

Gut microbiome: Gut bacteria regulate metabolic homeostasis by influencing processes such as glucose tolerance, insulin sensitivity, fat storage, hunger, and inflammation. A healthy intestinal microbial community can improve appetite, energy, adipogenesis, and thermoregulation.

Various animal studies have shown that feeding ASs to rats led to a decrease in the ratio of anaerobes to aerobes [30], notable augmentation in the mass of cecal contents, and a dose-dependent increase in the fecal content of soluble polysaccharides, leading to an increased availability of carbohydrates for the gut microbiota [31]. When ASs was used for more than 20 weeks, the average amount of ammonia in the cecal contents increased by 30-50%. At the same time, the activity of several bacterial enzymes decreased, which led researchers to think that this was one way ASs affected the gut microbiome [31]. ASs have been noted to alter the metabolism of amino acids by gut flora, resulting in the generation of carcinogenic substances. Additionally, researchers have postulated that saccharin has the potential to impede the process of intestinal protein digestion, resulting in heightened bacterial metabolism [32]. In an RCT comparing ASs (sucralose and maltodextrin) to a control group, *Bifidobacterium*, *Lactobacillus*, and *Bacteroides *levels were much lower in the AS group than in the control group. However, it did not demonstrate any discernible impact on enterobacteria. When sucralose and maltodextrin were used together, the pH of the feces increased, and the amount of P-glycoprotein and CYP450 enzymes in the intestines was higher [33].

Human studies performed by Suez et al. evaluated the impact of ASs on the human microbiome. A total of 381 individuals without diabetes who self-reported regular consumption of ASs, as determined by a food frequency questionnaire, were included. The study demonstrated a significant association between the consumption of ASs and the development of central obesity, elevated fasting blood glucose levels, increased hemoglobin A1c levels, impaired glucose tolerance, and elevated alanine aminotransferase levels. In addition, a subgroup analysis was conducted to compare those who consumed higher amounts of ASs with those who did not consume any ASs. The results of this analysis revealed a statistically significant elevation in hemoglobin A1c levels, even after controlling for body mass index. A total of 172 people were randomly selected from this cohort, and their intestinal microbial composition exhibited alterations, specifically marked by elevated levels of *Actinobacteria phylum*, *Deltaproteobacteria*, and *Enterobacteriaceae *[34].

Gastrointestinal motility: The potential impact of ASs on gastrointestinal motility is primarily mediated indirectly through its influence on the release of incretin hormones and serotonin. Several ASs have been found to induce elevations in the levels of cholecystokinin, which delays stomach emptying, and gastric inhibitory polypeptide, which may have an inhibitory effect on gastric emptying. ASs have also been demonstrated to increase glucagon-like-peptide-1 (GLP-1), which has been observed to reduce motility in the antro-duodeno-jejunal area and suppress the migrating motility complex in both individuals without any gastrointestinal disorders and those diagnosed with irritable bowel syndrome, and peptide YY (PYY), which can induce a delay in intestinal transit [35-39]. Multiple RCTs performed in humans have shown that ASs did not affect the secretion of GLP-1 or PYY [40,41], but interestingly, ASs did enhance GLP-1 release when given with glucose [42].

Anatomy of the gastrointestinal tract: The effects of ASs on the gastrointestinal tract, specifically gastrointestinal symptoms, gastrointestinal histology, anatomy of the gastrointestinal tract, and stool forms, have rarely been studied, with no human studies to date.

ASs have been noted to increase the stool water content by its osmotic effect [30], hyperkeratosis, papilloma, ulcers in the glandular stomach of rats [43], and DNA damage in the stomach and colon [44]. Histopathologic findings of the colon include infiltration of lymphocytes into the epithelium, scarring of the epithelial tissue, and a slight reduction in the number of goblet cells [33]. High doses of ASs (750-1,000 mg/kg/day) led to symptoms of perianal soiling and cecal enlargement in rabbits [45].

Intestinal absorption and permeability: There is limited data on the effect of ASs on intestinal absorption and permeability. Of the studies performed, they appear to inhibit the passive transport of sugar through the basolateral membrane, but this was not observed in a follow-up study by the same group [46,47].

Neurological manifestations: Most of the reports on the impact of AS on neurological manifestations come predominantly from studies of aspartame. For this section, aspartame will be used synonymously with AS. Aspartame, specifically, has been extensively implicated in triggering headaches. Other neuropsychological symptoms associated with aspartame include seizures, anxiety, depression, and insomnia.

Headaches and migraines: Aspartame is 55% phenylalanine and 45% aspartate. In contrast to dietary protein, aspartame consumption can increase brain levels of phenylalanine and aspartic acid. These compounds can inhibit the synthesis and release of known neurophysiological activity regulators, dopamine, norepinephrine, and serotonin. Aspartame functions as a chemical stressor by increasing plasma cortisol levels and triggering the production of excessive free radicals. High levels of cortisol and excess free radicals may increase the brain’s susceptibility to oxidative stress, which may have detrimental effects on neurobehavioral health [48].

Phenylalanine, an amino acid, is believed to play a role in the pathophysiology of migraines due to its participation in serotonin synthesis. Serotonin has the potential to exert an influence on the cerebrovascular alterations that are linked to the experience of pain in migraine headaches. Serotonin synthesis is contingent upon the presence of L-tryptophan, an essential amino acid, obtained from dietary proteins. Phenylalanine and L-tryptophan engage in a competitive process to obtain access to the brain, with limited availability. This competition is believed to be the primary factor contributing to a reduction in serotonin levels within the brain. The observed decrease in serotonin levels is believed to induce vasodilation, which is hypothesized to be the underlying mechanism responsible for the manifestation of migraine pain [49].

RCTs comparing aspartame to placebo in patients with headaches have reported an increase in headache frequency with the continued use of aspartame [50,51]. In a survey-based study [10], 8.2% of the 171 consecutive patients reported aspartame as a precipitating headache.

Taste alteration: It is unknown whether exposure to non-nutritive sweeteners (NNS) alters human taste perception, but there is some evidence to support this possibility. There is an inverse relationship between NNS use and blood oxygen level-dependent responses in the amygdala and insula in response to sucrose [52]. Thus, it is conceivable that the altered activity in these regions of heavy NNS consumers reflects a reduction in afferent signaling and the perceived intensity of sweet stimuli [53].

Allergic reaction: Multiple sweetening agents have been associated with allergic reactions, including aspartame, xylitol, and erythritol. Aspartame is metabolized to formaldehyde, a component responsible for systemic reactions, including skin rashes and contact dermatitis [54-56]. Xylitol has been associated with severe allergies, including oral ulcers and skin reactions [57]. Erythritol has also been associated with urticarial reactions [12].

Cardiovascular and Stroke Risk

Cardiovascular diseases (CVDs) are the leading global cause of mortality [58]. The relationship between ASs and cardiovascular risk is complex and not entirely clear-cut. Some studies have suggested potential associations with adverse cardiovascular outcomes, while others have found no significant harm. The direct assessment of AS intake’s impact on hard endpoints, such as CVD risk, through RCTs, has been precluded by ethical considerations.

One such study, conducted within the NutriNet-Santé cohort [59], revealed associations between sugary drinks and artificially sweetened beverages and an increased CVD risk. Within this cohort, an overall elevated risk of CVD and cerebrovascular disease was linked to total AS intake. Specifically, aspartame consumption was associated with an increased risk of cerebrovascular events, while acesulfame potassium and sucralose were linked to a heightened risk of coronary heart disease. These findings collectively indicate that substituting ASs for added sugar may not confer any cardiovascular benefits [59].

Systematic reviews and meta-analyses [60,61] have also pointed toward direct associations between artificially sweetened beverages and CVD risk. Notably, the World Health Organization (WHO) 2022 report on the health effects of ASs highlighted associations between the consumption of beverages containing ASs, used as a proxy, and certain intermediate markers of CVD [62]. These markers encompass a modest increase in the unfavorable total cholesterol to high-density lipoprotein cholesterol ratio and an elevated risk of hypertension. Furthermore, the international health authority identified heightened CVD mortality and increased incidence of cardiovascular events and strokes associated with greater consumption of soft drinks containing ASs.

An additional noteworthy aspect pertains to the study conducted by Andersson et al., where they conducted a cross-sectional investigation into the impact of sugar-sweetened beverages and diet soda on cardiac remodeling among consumers [63]. Although the researchers duly acknowledged the influence of elevated body weight as a confounding factor, their findings revealed a notable association between soda consumption, particularly diet soda, and heightened left atrial dimensions and left ventricular mass in contrast to individuals who refrained from soda consumption [63]. However, it is important to note that prospective studies in this regard remain limited, and the level of evidence for these associations is still categorized as low by the WHO.

Type 2 Diabetes Mellitus

The incidence of diabetes mellitus has experienced a notable increase in recent years, primarily attributed to our dietary choices and sedentary lifestyles [64]. In a recent extensive population-based cohort study involving 105,588 French adults, the consumption of ASs was found to be associated with an elevated risk of type 2 diabetes mellitus (T2DM). Specifically, positive correlations were identified for various sweeteners, including total sweeteners, aspartame, acesulfame-K, and sucralose [2].

Several meta-analyses have explored the relationship between ASs and diabetes. The meta-analysis conducted by Azad et al. [65] revealed a positive association between ASs and T2DM risk. Similarly, Qin et al. [66] reported a direct link between ASs and T2DM. The most recent analysis, conducted by the WHO in 2022 [62], found a higher incidence of T2DM associated with ASs and tabletop sweetener consumption. Collectively, these findings present a compelling case against the widespread consumption of ASs as a safe alternative to sugar. Instead, they underscore the importance of targeting a reduction in the prevalence of sugary tastes within Western diets. In light of these data, it is advisable not to recommend the extensive use of ASs, emphasizing the need for a broader approach to reducing sugar intake in Western diets.

Cancer Risk

Multiple research studies have been conducted to assess the potential link between AS and cancer risk. One of the first studies to raise concern about ASs was conducted in 1977, which demonstrated an association between ASs and bladder cancer. Howe et al. showed that in a case-control study, there was a 1.6 risk ratio for every user of ASs to develop bladder cancer compared to individuals who had never used these sweeteners [67]. In further studies to assess the association between AS and bladder cancer, multiple studies found the absence of a similar association [68-74]. In fact, in a systematic review and meta-analysis, no correlation was found between ASs and any type of cancer (odds ratio (OR) = 0.91, 95% confidence interval (CI) = 0.75-1.11). Interestingly, this study found an inverse correlation between urinary system cancer risk and the use of ASs in women (OR = 0.76, 95% CI = 0.60-0.97) [16]. In another observational study, only frequent consumption of artificially sweetened beverages in postmenopausal women (i.e., more than one drink per day) may be associated with a higher risk of kidney cancer [74]. In addition, in a meta-analysis of prospective studies with approximately 4 million participants, the intake of ASs was not associated with any type of cancer incidence or mortality [75].

However, new findings in rodents demonstrate that aspartame may be a chemical carcinogen in rodents, and prenatal exposure may elevate cancer risk in rodent offspring [76]. Nevertheless, these results have not been shown in human studies. Hence, the FDA still affirms that all approved ASs are safe to consume without any association with cancer risk. Importantly, more research studies continue to evaluate the potential effects of ASs on different aspects of health (e.g., gut microbiota and insulin response), which may indirectly impact cancer risk. Hence, more studies with adequate power are needed to understand the effect of using ASs on the development of cancer and whether a dose effect may mediate this association.

## Conclusions

The use of ASs has been constantly increasing in recent years. Despite the various uses of ASs, many reports have indicated multiple side effects associated with their use. In our comprehensive review, we demonstrate that ASs can impact various functions of the gastrointestinal, neurologic, and cardiovascular systems. Although multiple studies associate ASs with increased cancer risk, the majority of recent research data, including systematic reviews and meta-analyses, show no link between the use of ASs and cancer risk. However, more long-term prospective studies are needed to better characterize the effect of ASs on human health.
